# Microbial Community Coalescence for Microbiome Engineering

**DOI:** 10.3389/fmicb.2016.01967

**Published:** 2016-12-06

**Authors:** Matthias C. Rillig, Alia Tsang, Julien Roy

**Affiliations:** ^1^Institut für Biologie, Freie Universität BerlinBerlin, Germany; ^2^Berlin-Brandenburg Institute of Advanced Biodiversity ResearchBerlin, Germany

**Keywords:** microbiome, host-mediated microbiome selection, engineering, community ecology, community coalescence

## Background

Microbiome engineering, and especially plant host-mediated microbiome selection (Mueller and Sachs, 2015), is a recently introduced set of methods by which microbial communities are selected in order to maximize certain fitness- or performance-related host functions, most typically of plants, but also for animals. In essence this is a phenomenological approach, acknowledging the holobiont concept of host-microbe associations (Rosenberg and Zilber-Rosenberg, 2016), implicitly encompassing a complex set of generally not very well-understood eco-evolutionary processes including microbial community shifts, (micro-) evolutionary changes and changes in physiology. Clearly, future research will be aimed at unraveling many of the component processes taking place during microbiome engineering, and therefore it is important to fully capture all processes potentially involved. One aspect that has so far not been considered as an underlying process of importance in microbiome engineering is community coalescence; yet, it is quite clear that community coalescence is an integral part of microbiome engineering approaches.

Community coalescence is a recently introduced concept (Rillig et al., 2015) denoting the encounter and interaction of entire microbial communities (i.e., the merging of previously separate networks of microbes), a circumstance that is not fully captured by existing theories such as metacommunity theory. In community coalescence, new biotic networks are formed, and also aspects of the abiotic environment may change due to mixing of the source environments. Community coalescence may naturally be quite frequent, and there are many scenarios for this process to occur in soils (Calderón et al., 2016; Rillig et al., 2016) and other environments, including animal hosts and aquatic systems. Despite this, these coalescent events have consequences for which we currently lack the theory and predictive tools.

## Where community coalescence comes into play in microbiome engineering

Microbiome engineering comes in many different forms (Mueller and Sachs, 2015) and depending on which pathway is pursued community coalescence can be a more or less prominent feature, but community coalescence is clearly implicitly included in the engineering process. In principle community coalescence could be a key factor occurring at three stages of microbiome engineering approaches: at the start of the engineering, during the selection of communities, and at the end, when the engineered communities are to be added to a target substrate. We mostly use the example of plant-associated microbiome engineering in the following, but concepts are transferable to other situations as well.

### Starter microbiomes

Microbiome engineering studies typically start with a diverse community, which may arise from mixing natural communities. For example, Panke-Buisse et al. (2015) used a mixture of soils from agricultural, forest, and grassland sites, in order to increase the diversity of soil microbes in the starting material. This mixing of the soil would constitute a coalescence event where previously separate microbial communities now encounter each other. These coalescence events would also in part give rise to some of the variability that is necessary for the subsequent selection of traits (e.g., flowering time) to occur. Here timing may be quite important: how much time passes between the initiation of the coalescence (i.e., the mixing of soils or other substrates) and the beginning of the interaction with the host may be of paramount importance. With the eventual outcome of coalescence expected to be a new equilibrium microbial community, it may be advantageous to minimize the time between mixing and plant host interaction to allow a wider range of community modules to be present, in order to act synergistically and to engage with the plant. Additional considerations may be the source of the coalescing communities, for example how divergent (taxonomically, functionally) they are. Dedicated studies should test if this divergence can be optimized, for example by mixing rhizosphere microbial communities from terrestrial plants with communities associated with aquatic plants if engineering for increased flood resistance is the goal.

Not all studies so far have used mixing, for example Swenson et al. (2000) used a single soil source as the starting point of one of their selection experiments. However, it stands to reason that these community coalescence events could be harnessed to enhance the outcome of the subsequent host-mediated selection. On the other hand, one could argue that microbial diversity is typically already high enough without mixing of communities at the onset of a microbiome engineering exercise. Avoiding a coalescence event at the beginning of the experiment may favor the persistence of certain community modules, which may be crucial for the task to be engineered.

The community coalescence concept also includes a possible mixing of the environments of the interacting communities (Rillig et al., 2015). In this context, investigation of the ways in which the nature of the initial inoculum substrate, for example whole soil vs. a soil extract, influences the final outcome of the coalescence event could provide guidance for optimizing microbiome engineering procedures.

### Mixed propagation of microbiomes

One pathway of propagation during community selection may involve mixing microbial communities that are obtained from different plant hosts, basically via pooling of replicates within a selection treatment line (for example by pooling rhizosphere soil samples from different plants in the same treatment: Swenson et al., 2000; Panke-Buisse et al., 2015; or by pooling water samples: Blouin et al., 2015). This constitutes a coalescence event as well, but perhaps one with less drastic consequences compared to the other two phases, because these source communities are likely to be relatively similar (and become more so as selection progresses). Nevertheless, intermediate coalescence events may be important to consider for the selection lines since they may lead to the loss or formation of unique combinations of microbes or community modules. Following this logic, coalescence events at this stage might slow the selection process by disrupting functionally important community modules; this may thus counteract the rapid engineering of a stable community with predictable function.

### Application of engineered communities

The third point where community coalescence will inevitably be important is during the actual application stage: the selected or engineered microbial communities would be used in a real-world setting, which rarely would be a sterile situation, i.e., the entire engineered community would then be added to a resident microbial community, constituting another community coalescence event. For example, this would occur when the engineered community is added to a field soil or another substrate. This is quite different from inoculation with a single isolate, since entire communities are the interactants.

In the first two cases community coalescence could potentially play a positive and very decisive role, in terms of providing unique communities as the raw material upon which host-mediated selection can then act. Additionally, as a matter of design coalescence events could be eliminated from the first two phases of microbiome engineering altogether if deemed disadvantageous. However, in the third case there is an unavoidable risk of the engineered community failing to interact with the resident community in a way that ensures that the major players or community modules are still active and can establish. There could also be deleterious unexpected effects that are important to study. For example, microbiome engineering will typically occur in highly controlled conditions, and final application of engineered microbiomes may take place in very dissimilar environmental circumstances (e.g., different nutrient availability, temperature, substrate conditions) than encountered during the engineering experiment. And as with coalescence of the starter microbiome, the environmental mixing aspect of community coalescence can potentially give insights to enhance success of the application of the engineered microbiome, such as modifications to carrier substrates (and thus the environment to which the community is initially exposed).

## Microbiome engineering to inform community coalescence-and *vice versa*

The current broad interest in microbiome engineering as an emerging, high-profile research field is a chance to concomitantly drive the development of our understanding of community coalescence. This would require a set of additional measurements; specifically, it would require monitoring of source and resultant (coalescent) communities from any mixing event, for example the mixing at the beginning of the engineering process, and ideally the longer-term stability of the community. Repeated high-throughput sequencing of phylogenetic markers would be required for assessing changes in community composition. Here it could be informative to measure the rate of module formation and describe communities using network structure indices such as modularity. Functional consequences (e.g., plant biomass or plant stress resistance) would be assessed as part of the monitoring of the engineering process and could be enriched by data from functional characterization of the microbiome, including metagenomics or community metabolomics, depending on the process to be engineered.

In providing the community and function data for studying outcomes of community coalescence from quite a range of different community types, spanning a range of community trait spaces, mixing ratios, and other conditions, this much-needed information could be used to develop ideas about community coalescence and to catalyze the development of theory (e.g., Tikhonov, 2016). We think this is possible in particular because community coalescence events can, in terms of their effects on the microbiome, be separated from other processes during the host-mediated microbiome selection when measurements are properly timed, i.e., take place right before and then after the coalescent event.

An important next step would involve shifting from mere monitoring of coalescence effects during microbiome engineering to the targeted manipulation of the coalescence events themselves. In the simplest case this would involve conducting factorial engineering experiments with and without coalescence events in the first two phases. Study complexity could be increased to also capture mixing ratios and modes, among other parameters. This deeper insight into community coalescence and its explicit representation in study design could result in a longer-term feedback, in turn benefiting the more targeted engineering of microbial communities. For example, development of rules about effects of mixing ratios (potentially of particular importance for the addition of engineered communities to resident substrate communities), the nature of the mixing (e.g., surface contact or wholesale mixing), the interaction of community modules and networks, or about the functional consequences of community-level trait interactions could be advanced. We should not miss this excellent opportunity to use an applied research focus to drive our improved understanding of the nature of microbial communities.

## Author contributions

MR drafted the initial manuscript, AT and JR contributed concepts and ideas. All authors commented on the final version of the manuscript.

### Conflict of interest statement

The authors declare that the research was conducted in the absence of any commercial or financial relationships that could be construed as a potential conflict of interest.
